# Expansion of Circulating T Follicular Helper Cells in Children with Acute Henoch-Schönlein Purpura

**DOI:** 10.1155/2015/742535

**Published:** 2015-09-28

**Authors:** Jue Xie, Yan Liu, Lei Wang, Guoxiang Ruan, Huiming Yuan, Hong Fang, Jianyong Wu, Dawei Cui

**Affiliations:** ^1^Department of Blood Transfusion, The First Affiliated Hospital, School of Medicine, Zhejiang University, Hangzhou, Zhejiang 310003, China; ^2^Department of Dermatology, The First Affiliated Hospital, School of Medicine, Zhejiang University, Hangzhou, Zhejiang 310003, China; ^3^Kidney Disease Center, The First Affiliated Hospital, School of Medicine, Zhejiang University, Hangzhou, Zhejiang 310003, China; ^4^Department of Clinical Laboratory, The First Affiliated Hospital, School of Medicine, Zhejiang University, Hangzhou, Zhejiang 310003, China

## Abstract

Henoch-Schönlein purpura (HSP) is a common systemic small vessel vasculitis in children with disorder autoimmune responses. T follicular helper (TFH) cells play crucial roles in regulating immune responses. The aim of our study was to investigate the probable role of TFH cells in the pathogenesis of children with HSP. In this study, the frequency of circulating CXCR5^+^CD4^+^TFH cells with inducible costimulator (ICOS) expression in the children with acute HSP was significantly higher than that in healthy controls (HCs) but not CXCR5^+^CD4^+^TFH cells with programmed death-1 (PD-1) expression. Moreover, serum levels of IL-21 and IL-6 cytokines, IgA, and C3 in HSP children were also significantly higher than those in HCs. A positive correlation was observed between the frequencies of circulating ICOS^+^CXCR5^+^CD4^+^TFH cells and the serum IL-21 or IgA levels of acute HSP children, respectively. Additionally, the mRNA expression levels of interleukin- (IL-) 21, IL-6, and transcriptional factors (B-cell lymphoma-6, Bcl-6) were also significantly increased in peripheral blood from acute HSP children compared to HCs. Taken together, these findings suggest that TFH cells and associated molecules might play critical roles in the pathogenesis of HSP, which are possible therapeutic targets in HSP children.

## 1. Introduction

Henoch-Schönlein purpura (HSP), characterized by palpable purpura, gastrointestinal bleeding, arthritis or arthralgia, glomerulonephritis, and acute abdominal pain, is the most small vessel vasculitis in children [1, 2]. The annual incidence of HSP is approximately 1 per 5000 children aged ≤20 years, and it decreases with age, and more than 90% of children with HSP are below 10 years of age, and the peak incidence occurs at 4–6 years [3, 4]. HSP is a systemic inflammatory disease with disorder autoimmune response [1]. Several inflammatory cytokines such as interleukin- (IL-) 17, IL-10, IL-6, and transforming growth factor- (TGF-) *β* elevated serum immunoglobulin A (IgA) concentration and small vascular deposition of IgA-related immune complexes are involved in the pathogenesis of HSP [5–7]. Additionally, IgA-producing B-cell and CD4^+^ helper T (Th) cells as Th17, Th2, and Treg cells play critical roles in HSP [8–10]. However, the role of a new CD4^+^Th cell subtype named T follicular helper (TFH) cell closely associated with the production of IgA is completely unclear in the pathogenesis of children with HSP.

TFH cells are characterized by the expression of molecules such as chemokine (CXC motif) receptor 5 (CXCR5), inducible costimulator (ICOS), programmed death-1 (PD-1), interleukin- (IL-) 21 receptor (IL-21R), and transcriptional factors as Bcl-6 and so on [11–14]. Additionally, High-level IL-21 secretion is a distinctive characteristic of TFH cell [15, 16]. TFH cells play an important role in regulating B-cell responses that can produce specific antibodies such as IgA, IgG, and IgM in autoimmune diseases, infectious diseases, and tumors [16–18]. Moreover, circulating TFH cells have been characterized by ICOS^+^CXCR5^+^CD4^+^ and/or PD-1^+^CXCR5^+^CD4^+^TFH cells in previous reports [19, 20].

According to these findings, we hypothesized that circulating TFH cells may play a critical role in regulating the production of IgA, IgG, and IgM antibodies which mediated the pathogenesis of children with acute HSP. Thus, we explored the role of circulating TFH cells in the pathogenesis of children with acute HSP. We found that the frequencies of circulating CXCR5^+^CD4^+^TFH cells and ICOS^+^CXCR5^+^CD4^+^TFH cells but not PD-1^+^CXCR5^+^CD4^+^TFH cells in acute HSP children were significantly higher than those in HCs. Moreover, elevated serum IL-21 and IgA concentrations were closely correlated with the frequencies of circulating ICOS^+^CXCR5^+^CD4^+^TFH cells in acute HSP children, although the significant correlation was not found between increased serum IL-6 and C3 levels and frequencies of circulating CXCR5^+^CD4^+^TFH cells, ICOS^+^CXCR5^+^CD4^+^TFH cells, or PD-1^+^CXCR5^+^CD4^+^TFH cells, respectively. Furthermore, the expression levels of IL-21, IL-6, and Bcl-6 mRNA in peripheral blood from children with acute HSP were notably higher than those in HCs. These findings suggest that expanded frequencies of circulating TFH cells might play an important role in the pathogenesis of acute HSP children.

## 2. Materials and Methods

### 2.1. Children Demographics

Based on the modified criterion of Henoch-Schönlein purpura in 2008 [21], the clinical and laboratory data of 22 children with acute HSP in the Kidney Disease Center and dermatological department and 12 HCs who were well matched for age and sex were enrolled at the First Affiliated Hospital, School of Medicine, Zhejiang University. All the acute HSP children did not have any other diseases during the recent 3 months. None of the HCs (none of their family members) had a history of vascular or autoimmune diseases. All the samples were obtained from the children with acute HSP. In addition, written informed consent was obtained from all individuals according to the Declaration of Helsinki (1964), and the local Medical Ethics Committee of the First Affiliated Hospital, School of Medicine, Zhejiang University, approved the study.

### 2.2. Cell Isolation and Flow Cytometric Analysis

Human fresh peripheral blood samples of all individuals were obtained from the children with acute HSP and HCs. Peripheral blood mononuclear cells (PBMCs) isolated by density gradient centrifugation with Ficoll-Hypaque solution (CL5020, CEDARLANE, Netherlands) were transferred to sterile tubes and washed twice with phosphate-buffered saline (PBS). Subsequently, human CD4^+^T cells were isolated by human CD4^+^T cell isolation kit (number 130-096-533) (Miltenyi Biotec GmbH, Germany). Additionally, human PBMCs were stained with FITC-Mouse Anti-Human CD4 (BD Biosciences, San Diego, CA, USA), clone name: RPA-T4, isotype control: FITC Mouse IgG2b, *κ*, clone name: eBMG2b; PE-Mouse Anti-Human CXCR5 Biotinylated Monoclonal Antibody (BioLegend, San Diego, CA), clone name: J252D4, isotype control: PE Mouse IgG2a, *κ*, clone name: eBM2a; APC-Anti-Human ICOS (CD278) (BD Biosciences, San Diego, CA, USA), clone name: ISA-3, isotype control: APC Mouse IgG2a, *κ*, clone name: MOPC-173; PerCP-Cy5.5 Mouse Anti-Human CD279 (PD-1) (BD Biosciences, San Diego, CA, USA), clone name: EH12.1, isotype control: PerCP-Cy5.5 Mouse IgG1, *κ*, clone name: MOPC-21. All the staining procedures were performed according to the manufacturers' protocols, and the stained cells were analyzed by a flow cytometer of FACSCalibur and CELLQUEST software (Becton Dickinson, Sparks, MD, USA).

### 2.3. Analysis of Serum Cytokines

Serum cytokines of IL-21 and IL-6 were measured in triplicate by enzyme-linked immune sorbent assay (ELISA; BioLegend, San Diego, CA, USA) according to the manufacturer's protocols. Briefly, 50 *μ*L of assay buffer and 50 *μ*L serum were added to the well and incubated at room temperature for 2 hours while shaking at 200 rpm. After washing the plate four times with wash buffer, 100 *μ*L of Human IL-21 Detection Antibody solution was added to each well and incubated at room temperature for 1 hour while shaking. After washing the plate four times, 100 *μ*L of Avidin-HRP solution was added to each well and incubated at room temperature for 30 minutes while shaking. After washing the plate five times, 100 *μ*L of Substrate Solution was added to each well and incubated for 30 minutes in the dark. Finally, stop the reaction by adding 100 *μ*L of Stop Solution to each well, and read absorbance at 450 nm within 30 minutes. The assay procedure of human IL-6 cytokine was similar to that of human IL-21 cytokine.

### 2.4. RNA Extraction and Real-Time PCR

To evaluate the mRNA expression levels of IL-6 and IL-21 genes, the total RNA of each sample in the PBMCs from the cases (*n* = 22) and HCs (*n* = 12) was extracted by TRIzol reagent (Invitrogen, Carlsbad, CA, USA). To analyze Bcl-6 mRNA expression, the total RNA of each sample in the CD4^+^T cells from the cases (*n* = 10) and HCs (*n* = 8) was also extracted by TRIzol reagent. Next, cDNA was synthesized by a reverse transcription reagent kit (Takara, Dalian, China) in accordance with the manufacturer's protocol. Real-time PCR was used in triplicate by Takara SYBR Supermix (Takara, Dalian, China) depending on ABI 7500 analysis system (Applied Biosystems, Foster City, CA, USA). The amplification conditions were as follows: 5 min at 95°C for denaturation and then 40 cycles at 95°C for 10 s and 60°C for 40 s. The fluorescence values were collected at 60°C. The primer sequences were as follows [22]:

 IL-21: forward, 5′-CACAGACTAACATGCCCTTCAT-3′; reverse, 5′-GAATCTTCACTTCCGTGTGTTCT-3′.
 IL-6: forward, 5′-AGACAGCCACTCACCTCTTCAG-3′; reverse, 5′-TTCTGCCAGTGCCTCTTTGCTG-3′.
 Bcl-6: forward, 5′-CATGCAGAGATGTGCCTCCACA-3′; reverse, 5′-TCAGAGAAGCGGCAGTCACACT-3′.

Each gene was standardized by the expression of glyceraldehyde 3-phosphate dehydrogenase (GAPDH) with the following primers: forward, 5′-GTCTCCTCTGACTTCAACAGCG-3′; reverse, 5′-ACCACCCTGTTGCTGTAGCCAA-3′.

The data were analyzed by ABI 7500 software (Applied Biosystems, Foster City, CA).

### 2.5. Statistical Analysis

An overall variation among the different groups was analyzed by one-way ANOVA analysis. Data are presented as the means ± standard deviation (SD), and Student's unpaired or paired* t-*test was appropriately chosen between groups. Mann-Whitney *U* test was performed between the two studied groups for nonparametric data. Correlations between variables were analyzed by Spearman's correlation coefficient. A value of *p* < 0.05 was considered statistically significant. The data were analyzed by GraphPad Prism 5 software (GraphPad Software, Inc., San Diego, CA).

## 3. Results

### 3.1. Characteristics of Children with Acute HSP

**Figure 1:**
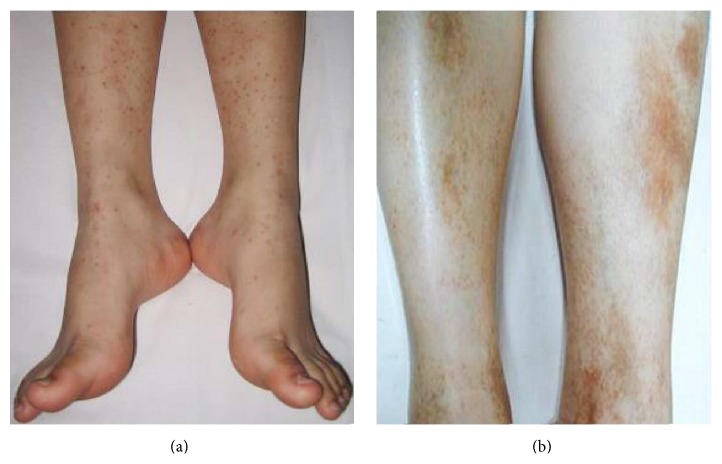
Cutaneous characteristics of children with acute HSP. (a) Cutaneous purpura of lower limbs of 8-year-old children. (b) Cutaneous purpura of lower limbs of 12-year-old children.

The clinical characteristics and laboratory parameters of the children with acute HSP and HCs are presented in Table 1. A total of 22 children (mean age, 8.27 ± 2.57 years) with acute HSP and 12 healthy children (mean age, 8.08 ± 2.47 years) as controls were recruited in this study. All children (100%) had cutaneous palpable purpura lesions (Figures 1(a) and 1(b)), 9 children (40.9%) had abdominal pain symptoms, 6 children (27.3%) and 1 children (4.5%) had gastrointestinal bleeding and renal involvement manifestations during the acute stage of HSP, respectively. Moreover, WBC count (*p* < 0.0001), serum IgA (*p* = 0.0081), and C3 (*p* = 0.0044) levels were significantly higher during the acute stage of HSP than the values in the HCs group. However, there were no significant differences about serum IgG (*p* = 0.1169), IgM (*p* = 0.6523), and C4 (*p* = 0.2628) levels between the children with acute HSP and HCs group (Table 1).

### 3.2. Expanded Frequency of Circulating TFH Cells in Children with Acute HSP

**Figure 2:**
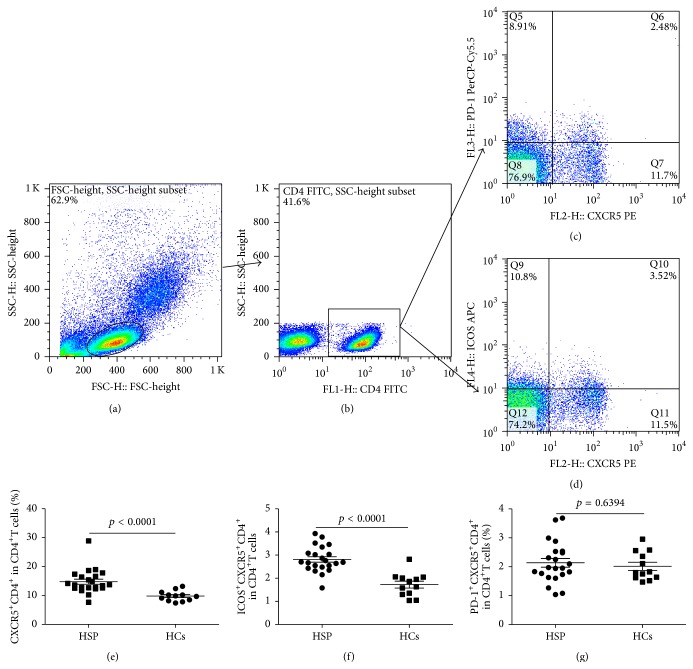
Increased percentages of circulating CXCR^+^CD4^+^TFH cells in the peripheral blood of HSP patients. Peripheral blood mononuclear cells (PBMC) from 22 patients with ITP and 12 healthy controls (HC) were isolated and stained with labeled antibodies and analyzed by flow cytometry as described in Section 2. The cells were gated initially on lymphocytes (a) and then on CD4^+^T cells (b). (c) ICOS^+^CXCR5^+^TFH cells in the total CD4^+^T cells of HC and HSP patients. (d) PD-1^+^CXCR5^+^TFH cells in the total CD4^+^T cells of HC and HSP patients. (e), (f), and (g) The analysis of percentages of CXCR5^+^CD4^+^T cells, ICOS^+^CXCR5^+^CD4^+^T cells, and PD-1^+^CXCR5^+^TFH cells from HCs and HSP patients. Each data pointer presents an individual subject. The horizontal lines show means.

**Figure 3:**
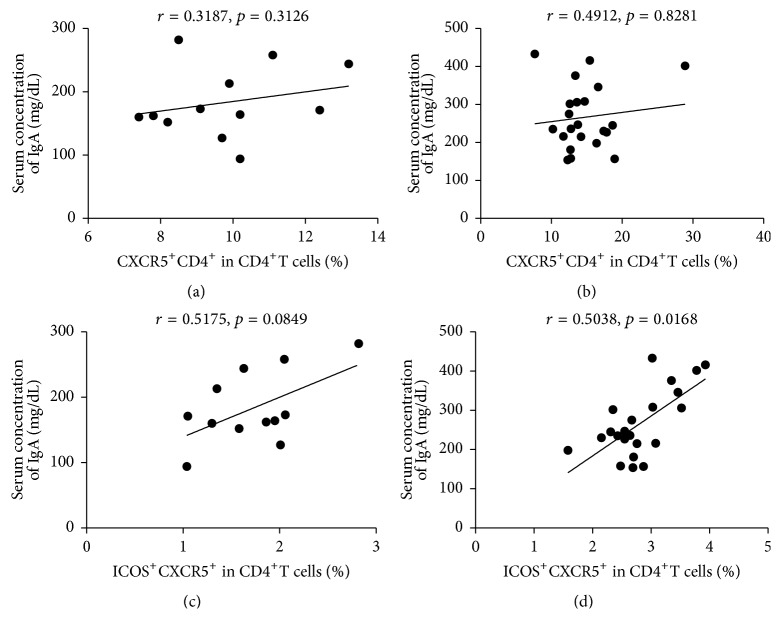
Correlation of serum IgA levels and circulating TFH cells in HCs and HSP patients. (a) and (b) Relationship of serum IgA levels and the percentage of CXCR5^+^ in CD4^+^TFH cells in HC and HSP patients, respectively. (c) and (d) Relationship of serum IgA levels and the percentage of ICOS^+^CXCR5^+^ in CD4^+^TFH cells in HCs and HSP patients, respectively. Data shown were the mean ± SD. The horizontal lines show the median.

To investigate the potential role of circulating TFH cells in children during the acute HSP, the frequencies of circulating CXCR5^+^CD4^+^TFH cells, ICOS^+^CXCR5^+^CD4^+^TFH cells, and PD-1^+^CXCR5^+^CD4^+^TFH cells were analyzed by flow cytometry (Figure 2). We analyzed CXCR5^+^T cells gated on CD4^+^T cells from human PBMCs to delimit circulating CXCR5^+^CD4^+^TFH cells in peripheral blood from the children with acute phage of HSP and HCs group (Figures 2(a)–2(d)). The percentages of circulating CXCR5^+^CD4^+^TFH cells and ICOS^+^CXCR5^+^CD4^+^TFH cells in the children with acute HSP were significantly higher than those of HCs, respectively (Figures 2(e) and 2(f)). However, the percentages of circulating PD-1^+^CXCR5^+^CD4^+^TFH cells were not different between the children with acute HSP and HCs group (Figure 2(g)). Additionally, a modest and positive correlation between the frequency of circulating ICOS^+^CXCR5^+^CD4^+^TFH cells and serum IgA levels was observed in the children with acute HSP, but no difference was found in HCs group (Figures 3(c) and 3(d)). There was also no significant correlation between circulating CXCR5^+^CD4^+^TFH cells and serum IgA levels in the children with acute HSP and HCs group (Figures 3(a) and 3(b)).

### 3.3. The Correlation between Serum Cytokine Levels and the Frequencies of Circulating TFH Cells in Children with Acute HSP

**Figure 4:**
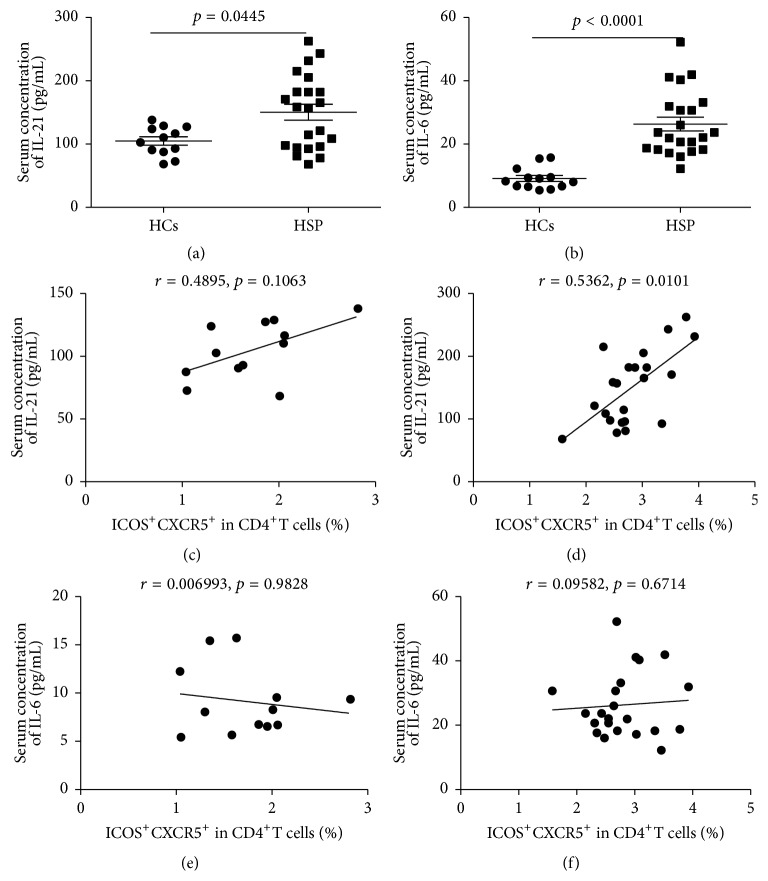
Correlation of cytokine levels and circulating TFH cells in HCs and HSP patients. (a) Levels of serum IL-21 in HCs and HSP patients. (b) Levels of serum IL-6 in HCs and HSP patients. (c) and (d) Relationship of serum IL-21 levels and the percentage of ICOS^+^CXCR5^+^CD4^+^TFH cells in HCs and HSP patients, respectively. (e) and (f) Relationship of serum IL-6 levels and the percentage of ICOS^+^CXCR5^+^ in CD4^+^TFH cells in HCs and HSP patients, respectively. Data shown were the mean ± SD. The horizontal lines show the median.

In order to explore TFH cells closely, associated cytokines levels in serum, serum IL-21, and IL-6 concentrations were measured by ELISA in serum from the children with acute HSP and HCs group (Figure 4). The serum IL-21 and IL-6 concentrations in children with acute HSP were significantly higher than those of HCs (Figures 4(a) and 4(b)). Moreover, the frequencies of ICOS^+^CXCR5^+^CD4^+^TFH cells were positively correlated with the serum IL-21 concentrations but not with serum IL-6 concentrations in the children with acute HSP (Figures 4(d) and 4(f)). Additionally, there were no correlation between the frequencies of ICOS^+^CXCR5^+^CD4^+^TFH cells and IL-21 or IL-6 levels in HCs (Figures 4(c) and 4(e)). Furthermore, the frequencies of CXCR5^+^CD4^+^TFH cells or PD-1^+^CXCR5^+^CD4^+^TFH cells were not significantly associated with serum IL-21 or IL-6 levels in the children with acute HSP or HCs (data no shown).

### 3.4. Expression of IL-21, IL-6, and Bcl-6 mRNA in Acute HSP Children

**Figure 5:**
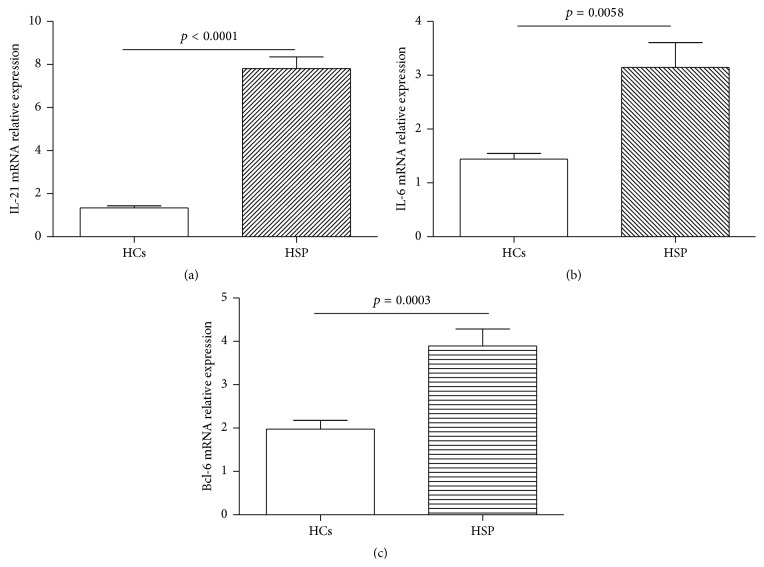
Expression of IL-21, IL-6, and Bcl-6 mRNA from HCs and acute HSP patients. (a) Levels of the relative expression of IL-21 mRNA in PBMCs from HCs (*n* = 12) and HSP patients (*n* = 22). (b) Levels of the relative expression of IL-6 mRNA in PBMCs from HCs (*n* = 12) and HSP patients (*n* = 22). (c) Levels of the relative expression of Bcl-6 mRNA in CD4^+^T from HCs (*n* = 8) and HSP patients (*n* = 10). Each data point represents an individual subject, and the data of each sample was an average value of three independent experiments with similar results. The horizontal lines show means.

Next, to further elucidate the role of TFH cells associated molecules, we detected the expressions of IL-21, IL-6, and Bcl-6 mRNA in HCs group and the patients with acute HSP (Figure 5). In this study, the results showed that the expression of IL-21, IL-6, and Bcl-6 mRNA in the children with acute HSP was higher than that of HCs, respectively (Figures 5(a)–5(c)).

## 4. Discussion

In this study, we found that the frequencies of circulating CXCR5^+^CD4^+^TFH cells and ICOS^+^CXCR5^+^CD4^+^TFH cells in peripheral blood obtained from the children in the acute phage of HSP were significantly higher than those of HCs group, although the frequencies of circulating PD-1^+^CXCR5^+^CD4^+^TFH cells was not significantly different between the children with acute HSP and HCs group. Moreover, the frequencies of circulating ICOS^+^CXCR5^+^CD4^+^TFH cells were positively associated with serum IL-21 or IgA levels in the children with acute HSP, respectively. Furthermore, serum concentrations of IL-21 and IL-6 cytokines, IgA, and C3 in the children with acute HSP were also significantly higher than those in HCs. Additionally, the expression levels of IL-21, IL-6, and Bcl-6 mRNA were also significantly increased in HSP children compared to HCs. In summary, these findings imply that ICOS^+^CXCR5^+^CD4^+^TFH cells and associated molecules may play crucial roles in the pathogenesis of children with acute HSP.

It is well known that HSP, nonidiopathic thrombocytopenic purpura (ITP), is the most common small vasculitis mainly caused by IgA-dominant immune complexes in children [1, 4, 21]. Diagnosis of HSP is commonly dependent on various clinical characteristics, such as cutaneous palpable rash, gastrointestinal bleeding, and arthritis or arthralgia [21]. Additionally, laboratory parameters are helpful for diagnosis of HSP [23]. In present study, clinical features such as mean age, cutaneous palpable purpura lesions, and gastrointestinal bleeding symptoms were in accordance with diagnostic criteria [21]. Moreover, notably increased serum IgA and C3 levels implied that they might be helpful for the pathogenesis of acute HSP, which were also in accordance with previous reports [24, 25].

Many studies have shown that the production of various immunoglobulins (Ig) such as IgA, IgM, and IgG is closely associated with TFH cells which play important roles in various autoimmune diseases such as ITP, rheumatoid arthritis (RA), systemic lupus erythematosus (SLE), and IgA nephropathy [22, 26–29]. However, the role of TFH cells remains unclear in the pathogenesis of HSP. The CXCR5 molecule is required for TFH cells migration to B cells follicular areas in germinal center (GC) and for the formation of GC [11–13]. Upregulation of CXCR5 can enhance the chance of antigen-specific contact between TFH cells and B cells in draining lymphoid tissue [13, 30]. Current results showed that increased frequencies of circulating CXCR5^+^CD4^+^TFH cells in peripheral blood from the children during the acute phage of HSP might be correlated with the pathogenesis of HSP compared to HCs. Regrettably, CXCR5^+^CD4^+^TFH cells were not detected in lymphoid tissues and/or spleen which were not obtained from children with HSP.

ICOS and PD-1 molecules are closely related with the function of CXCR5^+^CD4^+^TFH cells [31–35]. ICOS deficient in humans and mice are associated with reduction of CXCR5^+^CD4^+^TFH cells [31]. Moreover, ICOS/ICOS-L deficient mice cause remarkable reduction of antibody production and isotype switch and disturbed the formation of GC [31–33]. On the contrary, ICOS overexpression induces overproduction of CXCR5^+^CD4^+^TFH cells and causes exuberant GC responses and remarkably promotes antibody production with autoimmune disease in mice [13, 36]. PD-1 molecule is commonly considered to negatively regulate activated T cells, regulating function of B cells in GC [34, 35, 37]. Moreover, several studies have demonstrated that expanded frequencies of circulating ICOS^+^CXCR5^+^CD4^+^TFH cells and/or PD-1^+^CXCR5^+^CD4^+^TFH cells in various autoimmune diseases such as SLE, RA, ITP, and Sjögren's syndrome are positively associated with the autoantibodies production, TFH cells associated cytokines as IL-21, which aggravate the progression of those diseases [19, 25–28, 38]. In this study, we also observed that the frequencies of circulating CXCR5^+^CD4^+^TFH cells and ICOS^+^CXCR5^+^CD4^+^TFH cells were significantly increased in the children with acute HSP comparing to HCs. Moreover, the frequencies of circulating ICOS^+^CXCR5^+^CD4^+^TFH cells were positively associated with serum IgA concentrations in the children with acute HSP but not with serum IgG or IgM levels. Additionally, the frequencies of circulating PD-1^+^CXCR5^+^CD4^+^TFH cells were not significantly different between the children with acute HSP and HCs group, and the percentages of CXCR5^+^CD4^+^TFH cells and PD-1^+^CXCR5^+^CD4^+^TFH cells were not notably related with serum IgA, IgG, or IgM levels in acute HSP children or HCs, respectively. These findings implied that increased percentages of CXCR5^+^CD4^+^TFH cells and ICOS^+^CXCR5^+^CD4^+^TFH cells in acute HSP children might contribute to inducing IgA production which could cause the formation of IgA-containing immune complexes that promoted the development and progression of children during the acute stage of HSP.

Recent studies have demonstrated that IL-6 and IL-21 cytokines play critical roles in the differentiation, proliferation, and function of CXCR5^+^CD4^+^TFH cells and in response to antibodies [15, 16, 39]. IL-21 cytokine is required for germinal center formation, TFH cell proliferation and function, Ig-secreting B cells, and antibody response [15, 16]. Moreover, IL-6 can induce IL-21 secretion and TFH cell generation [39]. Additionally, activated CXCR5^+^CD4^+^TFH cells with ICOS and/or PD-1 expression can secret abundant IL-21 cytokine by themselves, and the levels of both IL-21 and IL-6 cytokines are significantly related with the frequency of circulating CXCR5^+^CD4^+^TFH cells with ICOS and/or PD-1 expression in some autoimmune diseases [17–19, 39–41]. In this study, there were significant differences about serum levels of IL-21 and IL-6 between HCs and the cases with acute HSP, which implied that they might play important roles in the pathogenesis of HSP disease. Moreover, increased serum IL-21 level is closely correlated with frequency of circulating ICOS^+^CXCR5^+^CD4^+^TFH cells in acute HSP children, which suggested that IL-21 plays predominant role in the generation and differentiation of TFH cells with ICOS molecule, which are consistent with previous study [39–42]. Bcl-6 transcription factor is required for the generation and differentiation of TFH cells, and Bcl-6 expression is regulated by IL-6 and IL-21 cytokines [14, 18, 36, 39]. PCR analyses demonstrated that the increased expressions of IL-21, IL-6, and Bcl-6 mRNA in children with acute HSP were possible explanations for the increased number of circulating CXCR5^+^CD4^+^TFH cells and ICOS^+^CXCR5^+^CD4^+^TFH cells in these acute HSP children.

However, in our study, any positive or negative correlation between cytokine levels or serum Ig levels and frequencies of TFH cells with ICOS and PD-1 expression were not observed in HCs, indicating that the balance of internal environment based on the balance of various cells and cytokines such as T cells, B cells, IL-21, and IL-6 in vivo contributes to the health of the body. Once the harmonious proportions of cells and cytokines are severely destroyed by internal or external factors such as pathogens and autoantigens, disease would occur such as HSP.

In summary, the results had shown that the frequencies of circulating CXCR5^+^CD4^+^TFH cells and ICOS^+^CXCR5^+^CD4^+^TFH cells, expressions of TFH cell associated molecules such as IL-21, IL-6, and Bcl-6, and serum IgA and C3 concentrations were significantly increased in the children during the acute phage of HSP compared to HCs, which implied that expanding the frequencies of circulating CXCR5^+^CD4^+^TFH cells and ICOS^+^CXCR5^+^CD4^+^TFH cells with increased IL-21 and IL-6 production might contribute to the production of IgA. Therefore, TFH cells and associated important molecules might play an important role in the pathogenesis of children with acute HSP, which would be new therapeutic markers for HSP disease in the future.

## Figures and Tables

**Table 1:** Clinical characteristics and laboratory data of children with acute HSP.

	HSP	HCs
Number	22	12
Age (years)	8.27 ± 2.57	8.08 ± 2.47
Sex (M/F)	13/9	7/5
Cutaneous palpable purpura	22 (100%)	N
Abdominal pain	9 (40.9%)	N
Gastrointestinal bleeding	6 (27.3%)	N
Renal involvement	1 (4.5%)	N
WBC (×10^9^)	10.40 ± 2.38	6.26 ± 1.12
Serum IgA (mg/dL)	266.50 ± 84.70	183.33 ± 55.30
Serum IgG (mg/dL)	1429.77 ± 528.59	1129.17 ± 273.19
Serum IgM (mg/dL)	173.86 ± 62.86	111.25 ± 33.66
Serum C3 (mg/dL)	135.29 ± 23.95	97.25 ± 18.94
Serum C4 (mg/dL)	37.43 ± 13.68	21.00 ± 6.28

Note: data are present as means ± standard deviation (SD). M/F: male/female; WBC: white blood cell; N: negative; HCs: healthy controls; HSP: Henoch-Schönlein purpura.
